# Contribution of Uncharacterized Target Genes of MxtR/ErdR to Carbon Source Utilization by Pseudomonas putida KT2440

**DOI:** 10.1128/spectrum.02923-22

**Published:** 2022-12-13

**Authors:** Tania Henríquez, Jyh-Shiuan Hsu, Jakob Sebastian Hernandez, Sonja Kuppermann, Michelle Eder, Heinrich Jung

**Affiliations:** a Ludwig-Maximilians-Universität München, Biozentrum, Mikrobiologie, Martinsried, Germany; Forschungszentrum Jülich GmbH

**Keywords:** carbon source utilization, *Pseudomonas putida*, acetate utilization, pyruvate, two-component regulatory systems

## Abstract

MxtR/ErdR is a two-component system that has been previously described as a regulator of the utilization of acetate in Vibrio cholerae and in some Pseudomonas species. Regulation is achieved by controlling the expression of the *acs* gene (acetyl-coenzyme A [CoA] synthetase). However, the physiological significance of other identified target genes is not fully understood. Here, we investigated the role of pp_0154 (*scpC*) and pp_0354/pp_0353 in the soil bacterium Pseudomonas putida KT2440. To this end, the genes were individually deleted and complemented in *trans*. Then, the growth of the resulting strains on different carbon sources was analyzed. To obtain information on protein function, a bioinformatic analysis was performed, and ScpC was purified and characterized *in vitro*. Our results indicated that *scpC* is important for P. putida KT2440 to cope with high concentrations of acetate. The encoded enzyme catalyzes the transfer of coenzyme A between acetate and succinate. On the contrary, pp_0353 and pp_0354 proved to be unimportant for the growth of the strain on acetate under our conditions. Extending the phenotypic analysis to other carbon sources led to the discovery that *mxtR*, *erdR*, and pp_0353 are important for the utilization of pyruvate as a carbon source. Taken together, the findings of this study expand the knowledge about the role of the MxtR/ErdR two-component system in carbon source utilization and about the specific functions of its target genes.

**IMPORTANCE** MxtR/ErdR and homologous two-component systems play important roles in the regulatory networks that control cell metabolism and influence bacterial-host interactions. Using the MxtR/ErdR two-component system of the plant growth-promoting soil bacterium Pseudomonas putida KT2440 as a model, this work elucidates the function of previously uncharacterized target genes of MxtR/ErdR and extends the knowledge of the physiological significance of the two-component system. Our results suggest that the target gene *scpC* encodes an acetate:succinate CoA transferase that is involved in the detoxification of acetate when it is present in large amounts. Furthermore, it is shown that MxtR/ErdR controls the metabolism of not only acetate but also pyruvate. This control involves the target gene pp_0353 (putative exonuclease). These findings may facilitate the optimization of P. putida KT2440 as a chassis for biotechnological applications and may contribute to a better understanding of the regulatory network of pathogens like Pseudomonas aeruginosa.

## INTRODUCTION

Acetate is a short-chain fatty acid that is secreted or used as a nutrient by various organisms. It is present in the environment, where its oxidation is taken as an indicator of organic carbon decomposition in anoxic sediments (1, 2), and it is also present in large quantities in the human intestine (between 30 to 100 mM) (3, 4). For years, it has been described that some bacteria, such as Escherichia coli, secrete acetate as a by-product of glucose consumption (overflow metabolism) (5). In this context, the accumulation of acetate in the medium, which is a less-preferred carbon source, inhibits bacterial growth. However, it has been reported that other organisms, such as Azotobacter vinelandii, prefer the consumption of acetate over glucose (6), indicating that the regulation of acetate metabolism is rather complex and varies among bacteria.

One of the strategies that microorganisms use to control the utilization of different carbon sources involves two-component systems (TCS). Activation by a physical or chemical stimulus results in the autophosphorylation of the sensor kinase (first component) and, after phosphotransfer to the response regulator (second component), in a cellular response (e.g., the activation of the transcription of target genes) (7). In Vibrio cholerae (8, 9) and in some Pseudomonas species (9–11), the utilization of acetate is under the control of the TCS MxtR/ErdR (also called CrbS/CrbR). Special features of the sensor kinase MxtR include an N-terminal, membrane-integral domain with similarities to the solute/sodium symporter (SSS) family and a solute carrier and TCS associated component (STAC) domain that links the transporter domain to additional domains that are typical for sensor kinases (10, 12, 13). The activation of MxtR by a yet unknown signal and the phosphorylation of ErdR stimulate the expression of *acs*. The gene encodes an acetyl-CoA synthetase that catalyzes the activation of acetate to acetyl-CoA (14). The acetyl moiety is then either completely oxidized via the tricarboxylic acid cycle or used for biosynthesis via the glyoxylate shunt (15). Another target gene of MxtR/ErdR that is associated with acetate utilization is *actP*, which is predicted to be involved in the uptake of acetate when the acid is present at low concentrations in the medium (10, 11). However, the contributions of further target genes (e.g., pp_0154 [*scpC*] and pp_0354) in Pseudomonas putida KT2440 to the utilization of acetate are unknown (10, 11).

In this context, we studied the role of *scpC* and pp_0353/pp_0354 in P. putida KT220 by analyzing the phenotypes of the respective deletion and complemented mutants. In addition, ScpC was purified and functionally characterized for the first time. Our results indicate that *scpC* encodes an acetate:succinate CoA transferase (ASCT). In parallel, our results show for the first time that MxtR/ErdR is important for pyruvate utilization, probably through its target genes pp_0353 and *actP-I* (pp_1743). Taken together, the findings of this study broaden the knowledge about the target genes of MxtR/ErdR and about the role of this TCS in P. putida KT2440.

## RESULTS AND DISCUSSION

### ScpC supports growth in minimal medium with acetate as the carbon source.

**FIG 1:**
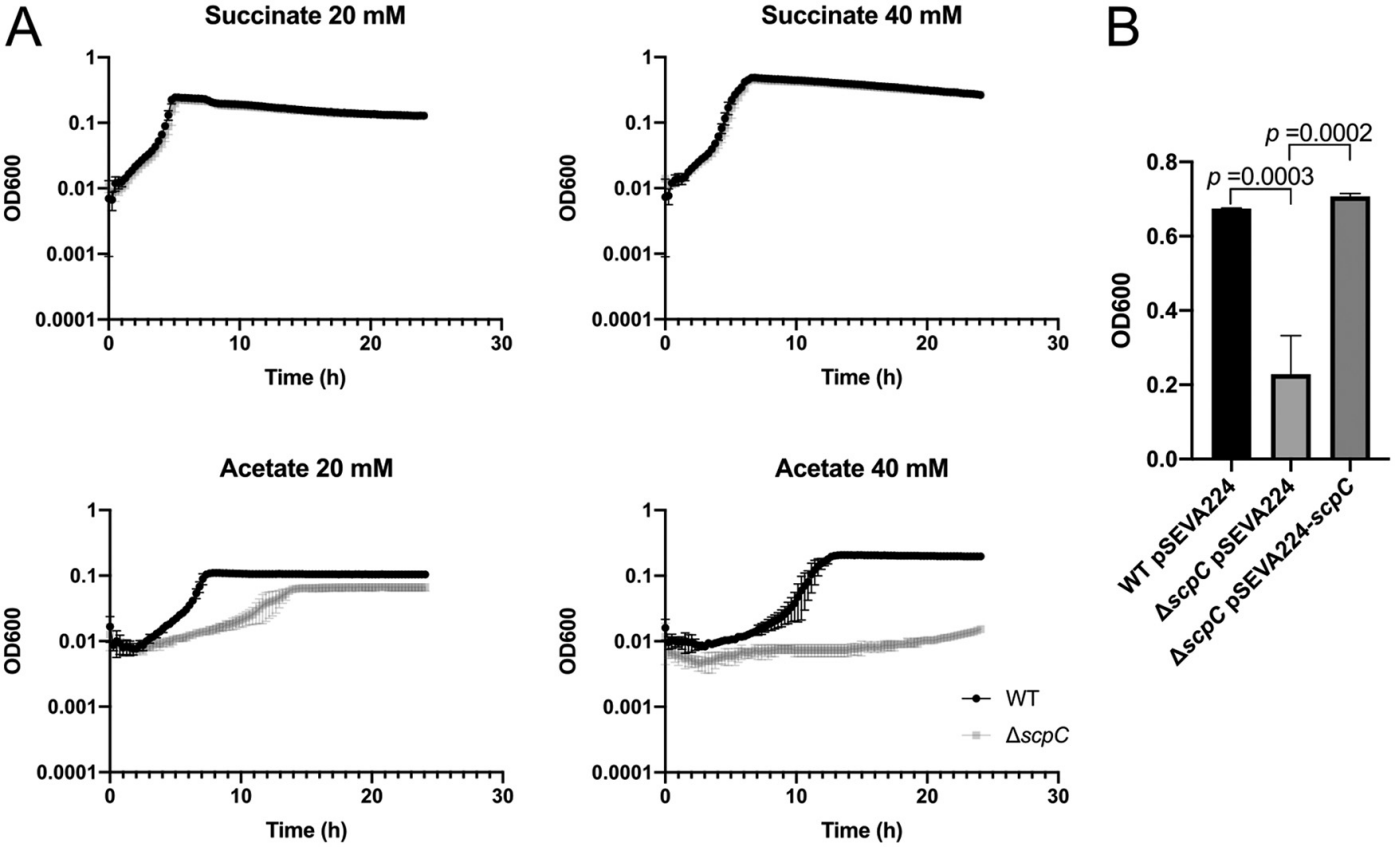
Growth analysis in minimal medium. (A) Growth curves of the WT and Δ*scpC* in minimal medium supplemented with 20 to 40 mM acetate or succinate. The experiments were performed in 96-well plates at 30°C with continuous shaking, and the OD_600_ was measured every 30 min. (B) The complementation experiments were performed in tubes containing 1 mL of minimal medium (20 mM acetate plus 0.5 mM IPTG) and incubated overnight (20 h) at 30°C with continuous shaking. All of the experiments were performed in triplicate.

Previously, we described the *in vitro* binding of the response regulator ErdR to the promoter region of the *scpC* of P. putida KT2440 (11). However, the function of the protein encoded by *scpC* has not been fully elucidated. Based on the significance of MxtR/ErdR for growth on acetate, we decided to analyze the role of *scpC* in acetate utilization. To that end, we generated a deletion mutant (Table 1) via homologous recombination and analyzed its growth compared to the wild-type (WT) strain in minimal medium supplemented with 20 mM succinate (control) or acetate as the carbon source. Our results showed that the growth of the Δ*scpC* mutant on acetate was reduced in comparison to the WT but was not affected when succinate was used as the carbon source (Fig. 1A). Since the Δ*scpC* mutant could still grow on 20 mM acetate, we decided to test whether higher concentrations of acetate would affect growth. Surprisingly, 40 mM acetate negatively affected the growth of the mutant, whereas the growth of the WT strain was improved, compared to 20 mM acetate (Fig. 1A), indicating that in the absence of *scpC*, higher concentrations of acetate are toxic for P. putida KT2440. To allow for the complementation of the defect in the Δ*scpC* background, we cloned *scpC* into the vector pSEVA224 Kan^R^ (16). The expression of *scpC* from the plasmid fully restored the growth of the Δ*scpC* mutant, compared to the WT strain (Fig. 1B). Taken together, these results indicate that in P. putida KT2440, *scpC* is important in coping with high concentrations of acetate.

**TABLE 1:** List of strains and plasmids used in this study

Name	Description	Source
Strains		
Wild-type (WT)	Pseudomonas putida KT2440	35
Δ*mxtR*	Derived from WT strain by deletion of pp_1695	11
Δ*erdR*	Derived from WT strain by deletion of pp_1635	11
Δ*scpC*	Derived from WT strain by deletion of *scpC*	This work
Δpp_0354	Derived from WT strain by deletion of pp_0354	This work
Δpp_0353	Derived from WT strain by deletion of pp_0353	This work
Plasmids		
pSEVA224	Km^R^; pSEVA221 derivative with *lacI^q^/Ptrc* expression system	16
pSEVA224-*scpC*	pSEVA224 derivative with *scpC* cloned into the multicloning site	This work
pSEVA224-pp_0353	pSEVA224 derivative with pp_0353 cloned into the multicloning site	This work
pET21a-*scpC*	pET21a derivative containing *scpC*	This work

### The gene *scpC* encodes an acetate:succinate CoA transferase.

**FIG 2:**
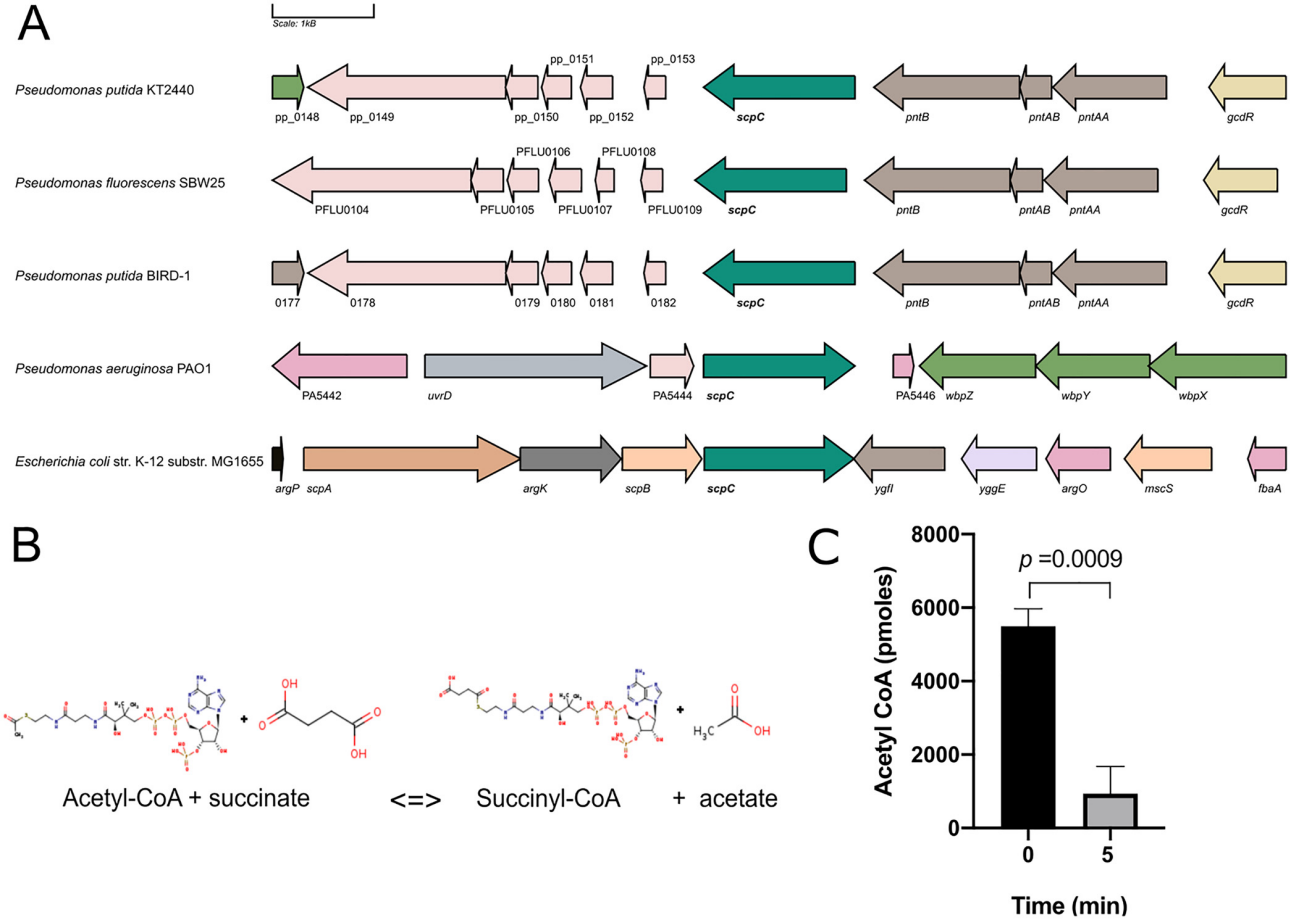
Genetic organization of *scpC* and analysis of the transferase activity of the gene product. (A) Diagram showing the genetic organization of *scpC* (bold) in different organisms. The figure was generated using the information available in the Pseudomonas Genome Database (https://www.pseudomonas.com/) and the Gene Graphics tool for visualization (https://www.genegraphics.net/app). (B) Scheme representing the reaction catalyzed by ScpC (modified and adapted from UniProt; https://www.uniprot.org/uniprot/B3EY95). (C) Determination of ScpC activity. The acetyl-CoA levels in the presence of succinate were measured in the absence of ScpC and 5 min after the addition of the enzyme. The incubation was performed at 25°C, and the levels of acetyl-CoA were measured using an Acetyl-coenzyme A Assay Kit (fluorometric test, Sigma). The experiment was performed in triplicate, and statistical significance was assessed via a *t* test using Prism 8 software.

**FIG 3:**
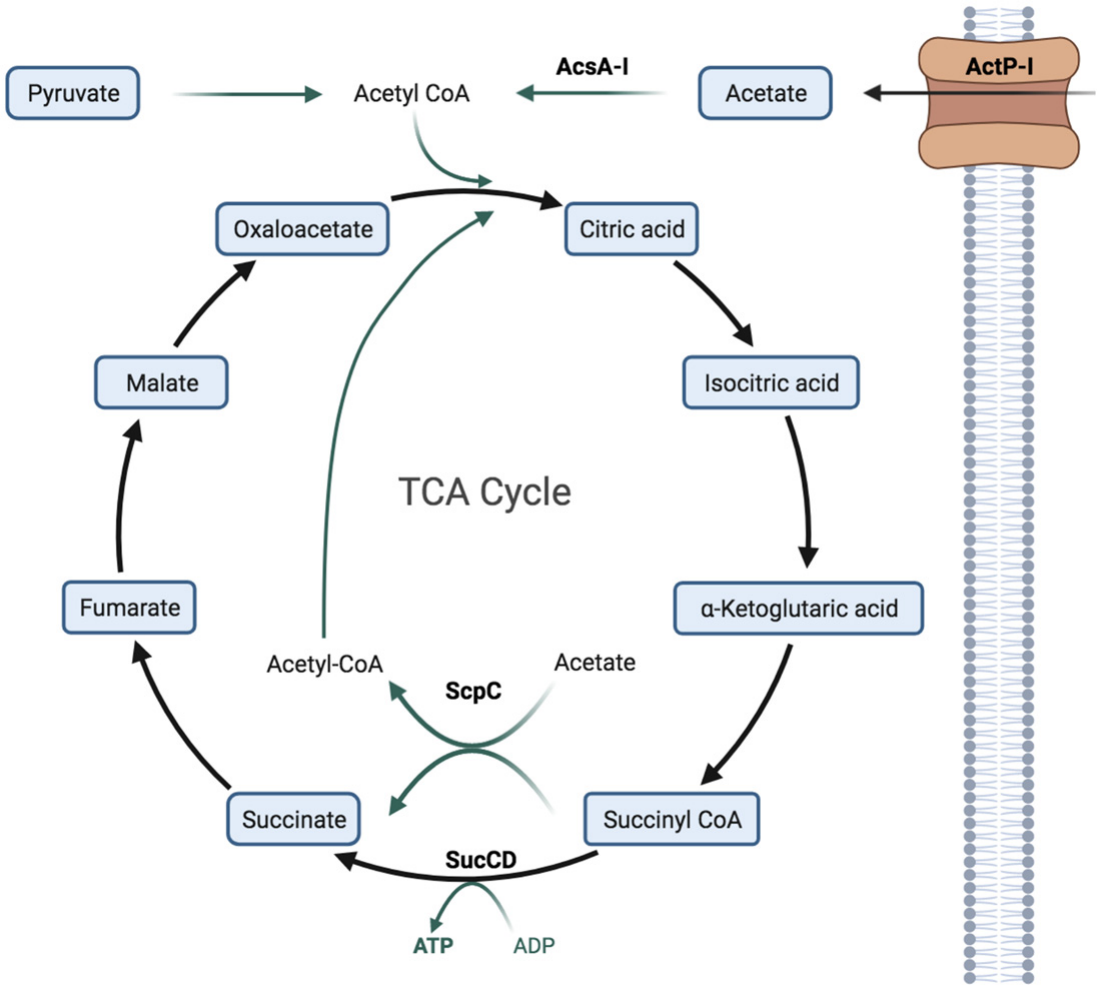
Visual representation of the role of products of target genes of MxtR/ErdR. The figure depicts the tricarboxylic acid (TCA) cycle of P. putida KT2440 as well as the proposed roles of the target genes of the MxtR/ErdR two-component system in the metabolism. ActP-I is predicted to function as an acetate transporter. The ActP homolog of E. coli transports acetate, pyruvate, and glyoxylate (34). AcsA-I is an acetyl-CoA synthetase that is involved in the activation of acetate. ScpC is an acetate:succinate CoA transferase that is involved in the utilization of acetate under high acetate conditions. Adapted from “Krebs Cycle” by BioRender.com (2022). Retrieved from https://app.biorender.com/biorender-templates.

Next, we searched for information on the role and genomic neighborhood of *scpC* homologs in other organisms. Our analysis revealed that in P. putida KT2440, P. putida BIRD-1, and P. fluorescens SBW25, *scpC* is located in a similar region between the operon encoding the pyridine nucleotide transhydrogenase and genes with unknown functions (Fig. 2A). However, in P. aeruginosa PAO1, *scpC* (also called *pseCoA*) is close to the gene *uvrD* (a DNA helicase II enzyme) as well as the genes *wbpZ*, *wbyY*, and *wbpX*, which have been linked to the assembly of the A-band and the d-rhamnan polysaccharide in this organism (17). In the case of E. coli, the homolog of *scpC* (also called *ygfH*) is located in an operon together with *scpA*, *argk*, and *scpB*, which, together with *scpC*, are predicted to code for components of a succinate decarboxylation pathway (18) (Fig. 2A). In all of these organisms, the homologs of *scpC* are predicted to be transferases. In the Pseudomonas Genome Database (19), the *scpC* of P. putida KT2440 is annotated as a propionate:succinate CoA transferase (https://www.pseudomonas.com/). In the Pfam database (https://www.ebi.ac.uk/interpro), ScpC is predicted to belong to the acetyl-CoA hydrolyze/transferase family. Based on this information and our previous results, we hypothesized that ScpC would most likely encode an acetate:succinate CoA transferase, which would be involved in the transfer of CoA between acetate or succinate (Fig. 2B). To test this hypothesis, we cloned *scpC* in a pET21a vector (attached to a sequence encoding a 6×His tag to the 3′ end of the gene) and expressed it in E. coli C43. Later, we purified ScpC via Ni-NTA affinity chromatography (Fig. S1) and determined its activity. To this end, we measured the reduction of the levels of acetyl-CoA in a mixture with succinate after 5 min of incubation (25°C) in the presence of ScpC. Our results indicated a significant reduction in the levels of acetyl-CoA (*P* = 0.0009). The enzyme activity for ScpC under the test conditions was calculated to be 893.2 nmol·min^−1 ^mg^−1^ (Fig. 2C). Additionally, we observed no significant reduction in the levels of acetyl-CoA when we performed the same experiment in the absence of succinate. With these results, we concluded that ScpC can transfer CoA between acetate and succinate, suggesting that ScpC can act as an acetate:succinate CoA transferase (ASCT). We assume that ASCT replaces succinyl-CoA dehydrogenase in the TCA cycle under acetate-rich conditions. It is likely that the role of ScpC *in vivo* is to activate acetate, using succinyl-CoA as a CoA donor without the need to hydrolyze ATP into AMP and pyrophosphate, as is done by acetyl-CoA synthetase (Fig. 3). It has been reported that this type of replacement represents a trade-off between nucleotide triphosphate synthesis via succinyl-CoA dehydrogenase (substrate-level phosphorylation) and the more efficient catabolism of acetate via an ASCT (20, 21). The utility of this enzyme for acetic acid assimilation and resistance was first demonstrated for a homolog of ScpC, namely, AarC of Acetobacter aceti (47.71% identity with ScpC of P. putida KT2440). The deletion of the *aarC* gene led to the inability of *A. aceti* to assimilate acetic acid (21). Later, Mullins and colleagues reported that *aarC* codes for an acetate:succinate CoA transferase that replaces succinyl-CoA synthetase, thereby generating a variant of the TCA cycle (22). For the homolog of ScpC in P. aeruginosa PAO1, a crystal structure is already available (PDB: 2G39), and it is predicted to bind acetic acid (23), which supports our findings.

Adding this new information on the role of MxtR/ErdR to the data previous collected by other groups, we propose a model to explain how this TCS controls acetate utilization: from one side, ActP-I allows for the uptake of acetate at low concentrations, while Acs and ScpC catalyze the conversion of acetate to acetyl-CoA in parallel, thereby allowing for it to be used in the TCA cycle and reducing acetate toxicity (Fig. 3).

### Impact of pp_0354 and pp_0353 in acetate utilization.

**FIG 4:**
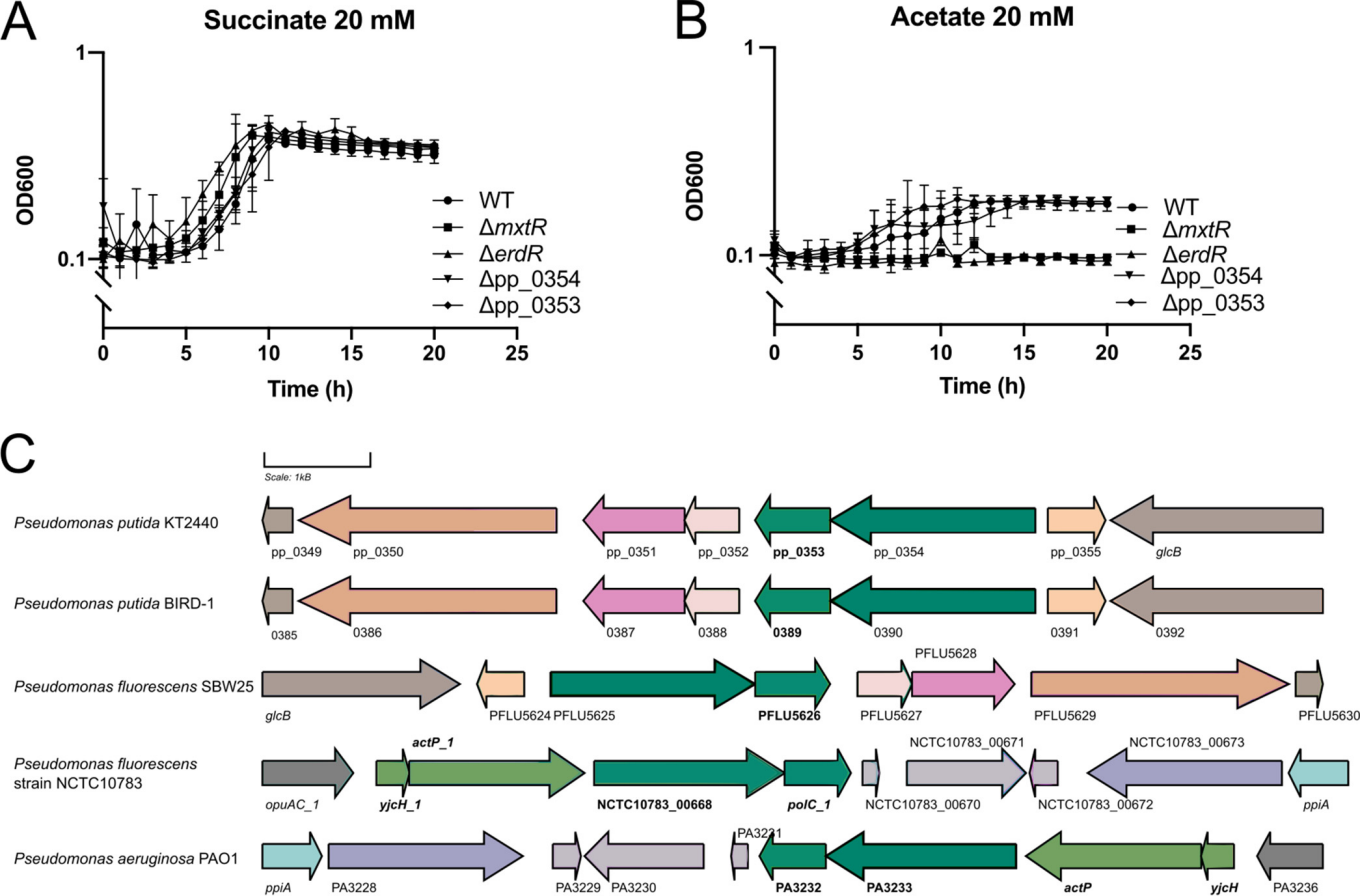
Growth analysis in minimal medium and the genomic neighborhood of pp_0353. Growth curves were performed in 96-well plates in minimal medium supplemented with 20 mM (A) succinate or (B) acetate. The experiment was carried out at 30°C with continuous shaking, and the OD_600_ was measured every 30 min. (C) Scheme showing the genetic organization of pp_0353 (bold). The scheme was generated using the information available in the Pseudomonas Genome Database (https://www.pseudomonas.com/) and the Gene Graphics tool for visualization (https://www.genegraphics.net/app).

In order to better understand the function of the target gene pp_0354 (a CBS domain-containing protein) of the MxtR/ErdR system, we decided to generate a deletion mutant for pp_0354 (for which we previously showed the direct binding of ErdR to its promoter region [11]). We then analyzed its growth. Our results did not reveal any growth defect when either 20 mM succinate or 20 mM acetate was used as the carbon source (Fig. 4A and B). Intrigued by this result, we examined the genetic neighborhood of this gene in P. putida KT2440 and found that pp_0353, downstream of pp_0354, forms an operon with pp_0354. To test the possible role of pp_0353 in acetate utilization, we generated a deletion mutant and analyzed its growth. Again, we observed a growth defect on neither succinate nor acetate (Fig. 4B). In order to obtain additional information on the function of these genes, we performed a bioinformatic analysis at the protein level and found a hit for pp_0353 for the RNase_T family in the Pfam database. Indeed, some homologs of pp_0353 in other organisms are annotated as 3′ to 5′ exonucleases. In this context, it has been described that some organisms can use nucleases to degrade exogenous DNA and can also use them as carbon or nitrogen sources when growing under limiting conditions (24). Although the amino acid sequence of pp_0353 does not contain any known secretion signal, we tested the growth of the mutant and the WT in minimal medium supplemented with 20 mM succinate or acetate in the presence or absence of DNA (0.2 g/L). Our results showed no difference in growth for any of the strains (Fig. S2). Furthermore, our strains did not grow in minimal medium supplemented with DNA alone, even at high concentrations (1 g/L), suggesting that pp_0353 is not involved in DNA consumption (Fig. S2).

Looking for other possible functions of pp_0353, we again checked the genomic neighborhood of pp_0353 and its homologs in related organisms, and we found that in P. putida KT2440, P. putida BIRD-1, and P. fluorescens SBW25, this gene is together with pp_0354 in a similar region and is close to the *glcB* gene (which codes for malate synthase G) (Fig. 4C). Interestingly, in P. aeruginosa PAO1 and P. fluorescens NCTC10783, the homologs of pp_0353 and pp_0354 are located next to *actP* and *yjcH*, which are other target genes of MxtR/ErdR, suggesting that their functions could be related. Indeed, the results of Rand and colleagues (25) using fitness assays with transposon mutants indicated that in P. putida KT2440, *actP-I* would be relevant for the growth of the strain in 5 mM acetate but not when higher concentrations are present, similar to the results that they reported for pp_0353. However, we were not able to investigate this finding in our lab because the WT and mutant strains did not grow on 5 mM acetate. Taken together, these results leave open the option that the predicted acetate transporter ActP-I and pp_0353 could have a role in acetate utilization under not yet identified environmental conditions.

### Role of pp_0353 in the utilization of pyruvate.

**FIG 5:**
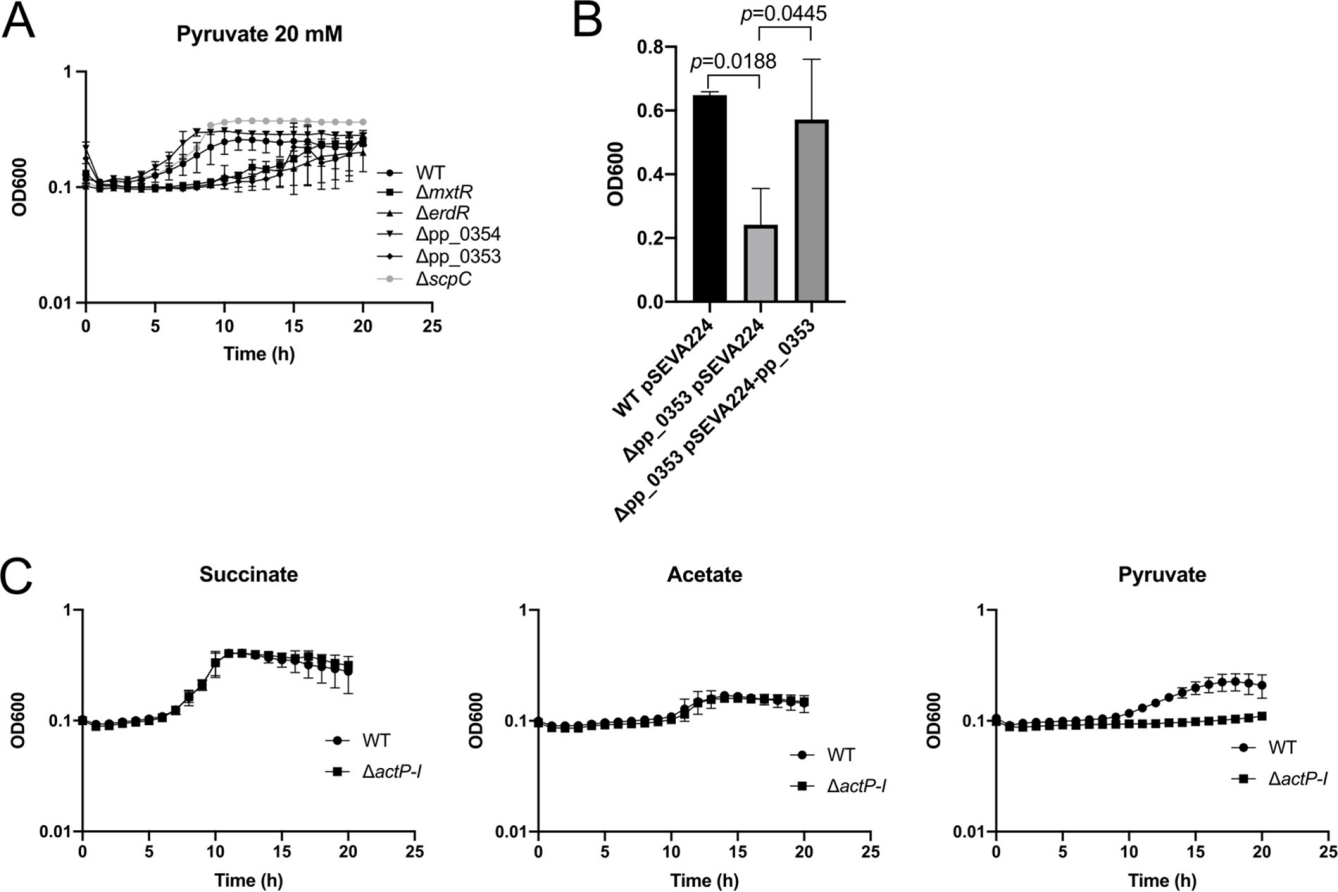
Growth analysis in minimal medium plus pyruvate. (A) Growth curves were performed in 96-well plates in minimal medium supplemented with 20 mM pyruvate. The experiment was carried out at 30°C with continuous shaking, and the OD_600_ was measured every 30 min. (B) The complementation experiments were performed in tubes at 30°C with continuous shaking. The OD_600_ was measured after 20 h of incubation. (C) Growth was analyzed in minimal medium supplemented with 20 mM succinate, acetate, or pyruvate. The experiments were performed in a 96-well plate format at 30°C with continuous shaking, and the OD_600_ was measured every 30 min. All of the experiments were performed a minimum of three times. The statistical analysis (*t* test) was carried out using Prism 8 software.

Other TCS with SSS- and STAC-containing sensor kinases, along with their target genes, control the catabolic pathways for amino acids and other organic acids (12, 26). In this context and without a phenotype for growth on acetate, we decided to test whether pp_0353 and pp_0354 (and finally, also MxtR/ErdR) are important for the utilization of other carbon sources. To this end, we tested the growth of these strains compared with the WT in minimal medium supplemented with 20 mM pyruvate and found that growth of the pp_0353 mutant, but not that of the pp_0354 mutant, was impaired (Fig. 5A). We also tested other mutants, such as the Δ*scpC*, Δ*mxtR*, and Δ*erdR* strains, and observed that the Δ*mxtR* and Δ*erdR* mutants, but not the Δ*scpC* strain, had a growth defect on this carbon source (Fig. 5A). This finding led us to wonder whether the role of MxtR/ErdR is broader than has previously been reported (11). Also, it is important to notice that the strains displayed delayed growth but did eventually reach the same OD_600_ as did the WT, indicating that MxtR/ErdR is not essential for pyruvate utilization. In contrast, if acetate was the carbon source, MxtR/ErdR was essential for growth (Fig. 4B). Similar to that observed with acetate, the growth defect of the mutants on pyruvate was fully complemented by expressing the genes from a plasmid (Fig. 5B). Also, following our previous findings about the possible link between the function of pp_0353 and the transporter ActP-I, we decided to test the growth of the Δ*actP-I* strain on pyruvate in comparison to acetate. Our results indicated that the *actP-I* mutant did not have a growth defect on 20 mM acetate (similar to the phenotype of the pp_0353 mutant), but, surprisingly, the Δ*actP-I* strain grew significantly worse than did the wild-type in minimal medium with 20 mM pyruvate (Fig. 5C). Taken together, these results indicate that the TCS MxtR/ErdR plays a role in pyruvate utilization, probably mediated by genes pp_0353 and *actP-I* as well as their products.

Taken together, the results show that, in contrast to *mxtR*, *erdR*, and *scpC*, the deletion of pp_0353 and pp_0354 had no impact on the growth of P. putida KT2440 on acetate. Instead, we discovered that pp_0353, and, consequently, the *mxtR* and *erdR* genes, are crucial for the utilization of pyruvate as a carbon source. In addition, we found that the deletion of the transporter gene *actP-I* impairs growth on pyruvate. These results are in agreement with our previous gene expression analysis, which demonstrated an upregulation of *actP-I* in cells grown on pyruvate, compared to cells cultivated on acetate (11). The mechanism behind this differential regulation of the expression of *actP-I* and the precise role of pp_0353 in pyruvate utilization are unclear. We speculate that the putative nuclease encoded by pp_0353 could help to degrade some of the mRNA of components of the TCA cycle, glyoxylate shunt, or transporters, similar to that which has been described in Corynebacterium glutamicum, in which a nuclease degrades the mRNA of *aceA* (isocitrate lyase) (27). However, we cannot exclude other mechanisms.

Interestingly, in addition to the MxtR/ErdR homolog CrbS/CrbR, a second TCS with an SSS/STAC-containing sensor kinase was recently reported in Sinorhizobium fredii NGR234 (26). This new system, designated RpuS/RpuR, proved to be important for pyruvate utilization by its upregulation of the *mctP* gene, which encodes a monocarboxylate transporter that, like ActP, belongs to the SSS family. In this context, we hypothesize that ActP-I is directly involved in pyruvate transport. Indeed, in other organisms, such as Rhodobacter capsulatus, ActP has already been linked to the uptake of acetate and pyruvate (28–30).

Taken together, this work expands the knowledge about the target genes of MxtR/ErdR and shows, for the first time, the involvement of this TCS in the utilization of pyruvate as a carbon source. These findings could be relevant for the optimization of the metabolic pathways and the biotechnological potential of P. putida KT2440.

## MATERIALS AND METHODS

### Bacterial strains and culture media.

A complete list of the strains and plasmids used in this study is available in Table 1. All strains were cultured in lysogenic broth (LB) and were maintained as frozen glycerol stocks. For experiments with different carbon sources (20 and 40 mM succinate or acetate as well as 20 mM pyruvate), minimal medium was prepared as previously described (11, 31), with small modifications: 1× M9-salts, 18.7 mM NH_4_Cl, 0.2 mM CaCl_2_, 2 mM MgSO_4_, and trace elements (134 μM Na_2_-EDTA, 31 μM FeCl_3_, 6.2 μM ZnCl_2_, 0.76 μM CuCl_2_, 0.42 μM CoCl_2_, 1.62 μM H_3_BO_3_, and 0.081 μM MnCl_2_). To test complementation, minimal medium was supplemented with 5 μg/mL kanamycin plus 0.1 mM IPTG. For the purification of ScpC, Escherichia coli strains were grown in LB supplemented with 100 μg/mL ampicillin.

### Generation of plasmids and mutants.

Knockout strains were generated via homologous recombination, as described previously, using the pNPTS138-R6KT suicide vector (32). A complete list of the primers used in this study can be found in Table S1. To this end, the upstream and downstream region of each gene was amplified via polymerase chain reaction (PCR). Later, the fragment was digested and ligated to the suicide vector. After checking the insert via sequencing, P. putida KT2440 was transformed with the plasmid (first recombination) and was grown on cetrimide plates containing 5% sucrose to screen for the second recombination. The final strain was confirmed via PCR and sequencing. For overexpression and complementation, each gene was amplified, cloned into the pSEVA224 vector (16), and used to transform the P. putida KT2440 wild-type strain or the corresponding mutant strain.

### Growth experiments.

Overnight cultures in LB were used to inoculate 100 μL of minimal medium supplemented with the corresponding carbon source (to get an initial OD_600_ of approximately 0.1) in a 96-well plate. We used LB for the overnight culture because there was no impact on the growth of the wild-type in comparison to minimal medium plus succinate in the subsequent main culture (Fig. S3). The growth curves were performed at 30°C with continuous orbital shaking (600 rpm) in a CLARIOstar plate reader. The complementation experiments were performed in tubes containing 1 mL of minimal medium supplemented with the corresponding carbon source plus 0.1 mM IPTG and 5 μg/mL kanamycin. The tubes were incubated with continuous shaking at 30°C, and the OD_600_ value was measured after 20 h.

### Protein purification.

The gene encoding *scpC* was cloned into pET21a in frame with a nucleotide sequence encoding a C-terminal 6× His tag and used to transform Escherichia coli C43. Briefly, an overnight culture grown at 37°C was used to inoculate 1 L of LB medium supplemented with 100 μg/mL ampicillin (initial OD_600_ of 0.1). The culture was incubated at 30°C with continuous shaking until an OD_600_ value of approximately 0.5 was reached. Then, induction was performed with 0.5 mM IPTG for 3 h. The pellet of the culture was later collected via centrifugation, washed once with 50 mM Tris-HCl buffer (pH 8), and then resuspended in lysis buffer (50 mM Tris-HCl [pH 7.5], 300 mM KCl, 5% glycerol, and 0.5 mM PMSF). For the cell disruption, the cells were processed using a Constant Cell Disruptor BT40/TS2/AA (Constant Systems Ltd.) (pressure: 1.35 kbar). The cellular debris were separated from the supernatant via low-speed centrifugation. Later, after high-speed centrifugation (44,000 rcf, 1 h) the supernatant was used for affinity chromatography with Ni-NTA. To this end, the sample was applied stepwise to the resin, which was packed in a column (Econo-Column, Bio Rad). The column was later washed with imidazole (10 and 30 mM). Finally, the protein (ScpC6H) was eluted in six fractions in buffer containing 50 mM Tris-HCl (pH 7.5), 300 mM KCl, 5% glycerol, and 200 mM imidazole. A Bradford assay was performed to determine the protein concentration. A Coomassie-stained SDS gel was prepared to estimate the purity of the protein, and a Western blot was performed in parallel to check the identity of ScpC6H. Hence, a mouse anti-6× His and a chicken anti-mouse HRP conjugated antibody were used (Fig. S1). To minimize the concern that the acetate:succinate CoA transferase activity that we detected was from an impurity that was not visible in the SDS-PAGE (Fig. S1), the SDS-PAGE and Western blot analyses were repeated with increasing amounts (2, 4, 8 μg) of purified ScpC (Fig. S4). In addition, size exclusion chromatography was performed using a Superdex 200 column (Fig. S5).

### ScpC transferase activity.

To determine transferase activity of ScpC, an Acetyl-Coenzyme A Assay Kit from Sigma (MAK039) was used, according to the manufacturer’s instructions. Briefly, 0.43 mM acetyl-CoA plus 20 mM succinate were mixed in an Eppendorf tube in the presence of 1× reaction buffer (50 mM Tris-HCl [pH 7.5], 100 mM KCl) in a total volume of 40 μL. From this original reaction, 20 μL were kept on ice (sample 0 min). Then, 1 μg of ScpC was added to the original reaction (total volume of 21 μL) and incubated for 5 min at 25°C (sample 5 min), after which 5 μL of 5 M perchloric acid (PCA) was added to both samples and incubated on ice for 5 min. The samples were then centrifuged at 10,000 rcf at 4°C and neutralized with 15 μL of 3 M potassium bicarbonate (added as 3 aliquots of 5 μL each). Then, the samples were cooled down on ice for 5 min, centrifuged for 2 min, and used to determine the acetyl-CoA levels. To that end, 3 μL of each final reaction were diluted with 47 μL of acetyl-CoA assay buffer and put into a 96-well plate with the reaction mix. For the standard curve, the 0 to 1 nmol range was used. All standard concentrations were tested in duplicate, and the samples were tested in triplicate, following the instructions of the manufacturers for the samples, standards, and blanks. Finally, the 96-well plate was incubated for 10 min at 37°C in a horizontal shaker (plate covered from light) and measured at 535 nm excitation and 587 nm emission in a Tecan Infinite M200 PRO plate reader. The obtained numbers were multiplied by the corresponding dilution factor and were used, together with the standard curve, to obtain the level of acetyl-CoA in each sample.

### Bioinformatic analysis.

For the analysis of *scpC*, pp_0353, and pp_0354, protein sequences were taken from the Pseudomonas Genome Database (https://www.pseudomonas.com/) and were used to search in the Pfam Database (https://www.ebi.ac.uk/interpro) and in the BLASTp suite. Similarly, DNA sequences were also taken from the Pseudomonas Genome Database and were used to analyze the genetic neighborhood with the Gene Graphics tool for visualization (https://www.genegraphics.net/app) (33). The DNA sequence from E. coli was obtained from the EcoCyc E. coli Database (https://ecocyc.org/).

### Statistical analysis.

The GraphPad Prism 8 software package was used for the statistical comparisons. As appropriate, either *t* tests or one-way analyses of variance (ANOVAs) with multiple comparisons were performed. For all of the graphs, the error bars represent the standard deviation of the corresponding values. All of the experiments were performed a minimum of three times.
