# Supplementary material for: Contribution of Uncharacterized Target Genes of MxtR/ErdR to Carbon Source Utilization by Pseudomonas putida KT2440
Source: Microbiol Spectr. 2022 Dec 13;11(1):e02923-22. doi: 10.1128/spectrum.02923-22 (PMC9927547; doi:10.1128/spectrum.02923-22)
Supplement: Supplemental file 1 — Supplemental material. Download spectrum.02923-22-s0001.pdf, PDF file, 0.5 MB [file spectrum.02923-22-s0001.pdf]

Supplemental material

Contribution of uncharacterized target genes of MxtR/ErdR to carbon source utilization by *Pseudomonas putida* KT2440

Tania Henríquez[a]#, Jyh-Shiuan Hsu[a], Jakob Sebastian Hernandez[a], Sonja Kuppermann[a], Michelle Eder[a], Heinrich Jung[a]#

[a] Ludwig-Maximilians-Universität München, Biozentrum, Mikrobiologie, Martinsried, Germany

Running Head: Characterization of target genes of MxtR/ErdR system.

# Address correspondence to Heinrich Jung, hjung@lmu.de; Tania Henriquez, thenriqueza@ug.uchile.cl

Table S1. List of primers used in this study.

Name	Sequence 5’-3’
Del scpC A_S	GCAGTAGTTTCAGTAGCCCG
Del scpC A_AS	GTCCACATTGATGGCTTTGC
Del scpC B_S	CAATGTGGACATCCTGGTCA
Del scpC B_AS	CTGTTATGGCCCATTCGCG
Clone scpC F	CGCCATATGTACCGTGATCG
Clone scpC R	GTCTAGAACAGCGGAAGCAG
Check scpC F	GCCTGGACAACGAACTG
Check scpC R	GCGTAACTGTACTGGTCCG
BamHI_optRBS_NheI_scpC_s	TTTGGATCCAAAGGAGGAAAAACATAAAGCTAGCATGTACCGTGATCGTATC
ScpC AS_XbaI	GTACCACATCTAGATCAGCTGGCCAG
Del0354_A_S	CAACATGGCCGGTACCTATA
Del0354_A_AS	TCGATGTTTGGCCAGGTGCT
Del0354_B_S	CAAACATCGAACCGTTGGACGG
Del0354_B_AS	TCACGCAGGGTACATACC
check0354F	ACAGTACCTGATCACGGTTG
check0354R	ATTCCGCTTCAGCATTAGGA
Delpp_0353 A_S	CGGAACGAATGCATTAACTA
Delpp_0353 A_As	GTCGAAGAACGCCATCATGC
Delpp_0353 B_S	GTTCTTCGACAGTACCTGAT
Delpp_0353 B_As	CTTACTCCAACCGGC
check pp_0353 F	GGCTTTGTGCAGAAGACAAG
check pp_0353 R	CCACTTCATCCAGCAAACCC
pp_0353 comp F2	ATGAGCCCTGTTCGCCTGG
pp_0353 comp R2	TGCTCTAGACGCTTATCGGATC
delpp_1743 A_S	GTCCTGAGTCATCGATTACC
delpp_1743 A_As	TGCTGACTTGTTGCGTTTGG
delpp_1743 B_S	CAAGTCAGCATCTGGTTCTT
delpp_1743 B_As	TCCTGGATAGCGAAGGTGAT
pp_1743 check F	TTCATCCTCCTGATCGCC
pp_1743 check R	TGTTGCTTGGGATGATATGA
scpC_EcoRI-3codons_as	GTACCACATGAATTCCAGCTGGCCAGC

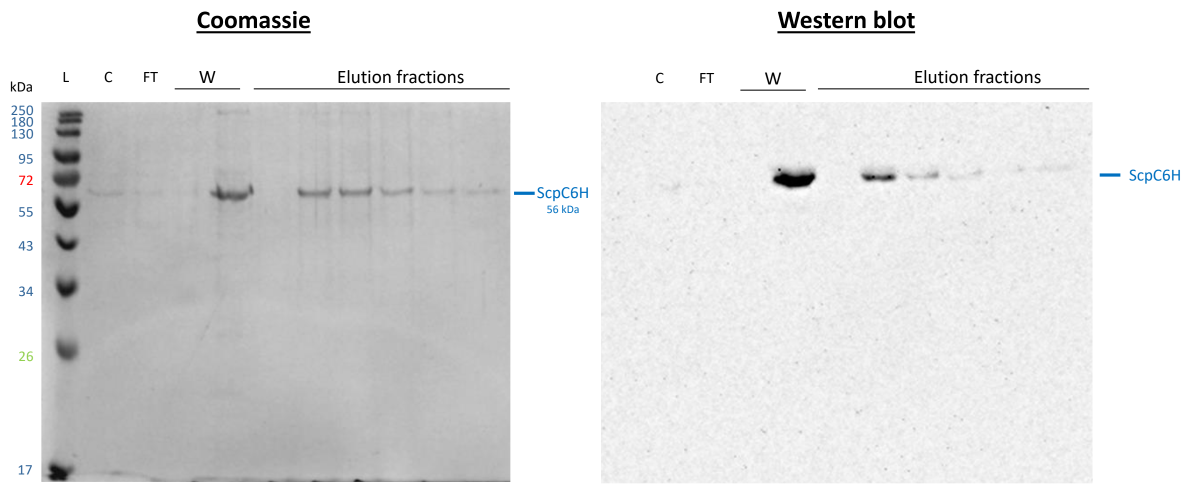

Figure S1. Purification of ScpC. The purity of ScpC was estimated via an SDS-gel stained with Coomassie and the identity by Western Blot (anti-6xHis and anti-mouse HRP conjugated antibodies). L: protein ladder, C: cytosolic fraction, F: flow through, W: wash steps. In total, 13.4 mg were obtained from cells of 1 l culture. The results of additional analyses of the puritiy of the ScpC preparation are shown in Figs. S4 and S5.

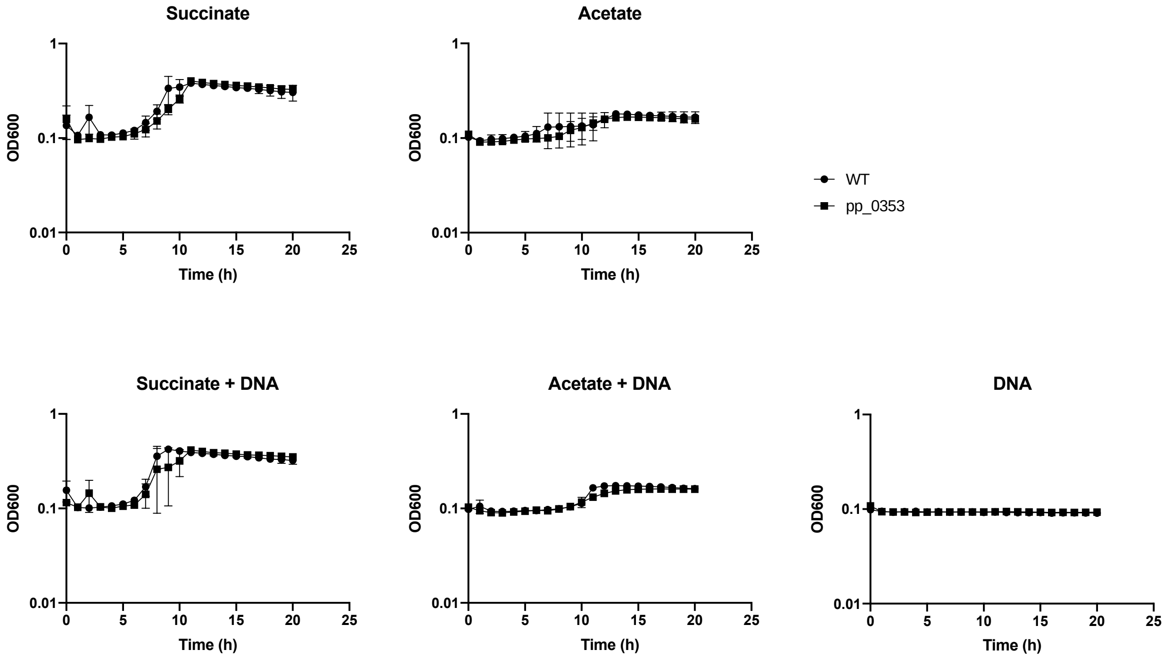

Figure S2. Growth of *P. putida* KT2440 and the derived pp_0353 mutant in minimal medium supplemented with DNA. Growth was analyzed in minimal medium supplemented with 20 mM succinate or acetate and 0.2 g/L DNA (shredded herring sperm). The experiments were performed in a 96-well plate at 30°C with continuous shaking and the OD600 was measured every 30 min.

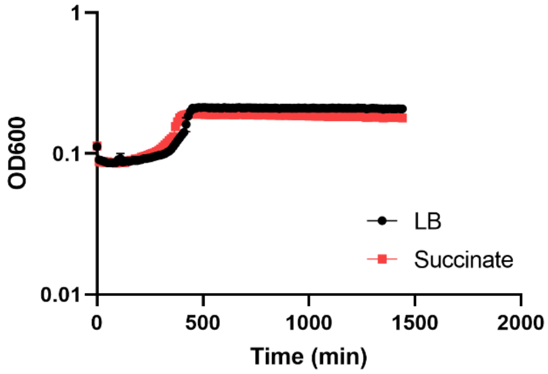

Figure S3. Effect of the type of medium used for pre-culture on growth. *P. putida* KT2440 was grown overnight in LB or M9 medium plus 20 mM succinate and then used to inoculate a new culture in M9 medium plus 20 mM acetate (OD 0.1). The experiments were performed in a 96-well plate at 30°C with continuous shaking and the OD600 was measured every 10 minutes. Shown are mean values of three biological replicates with two technical replicates per biological replicate.

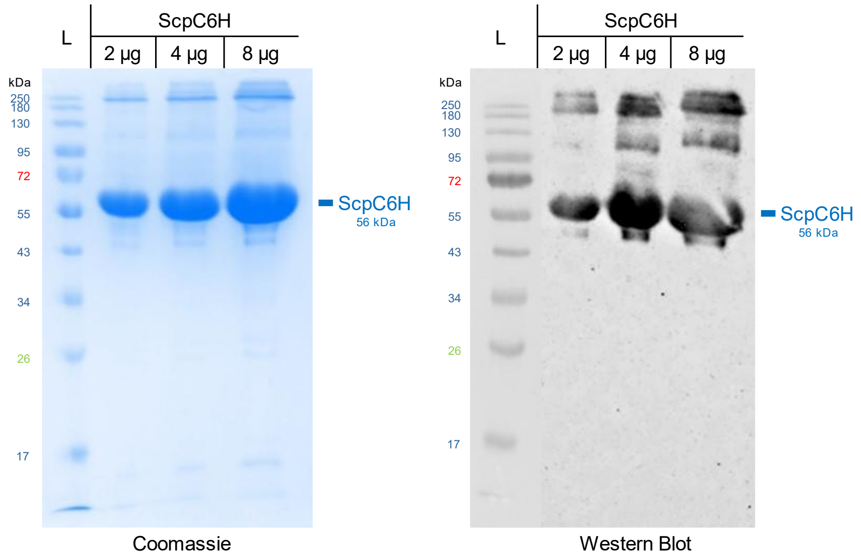

Figure S4. Analysis of the purity of ScpC. In addition to the analysis presented in Fig. S1, the purity of the ScpC preparation was further tested by applying 2, 4, and 8 µg of purified ScpC to SDS-PAGE. The resulting gel was stained with Coomassie (left panel) and the identity of the protein was determined by Western blotting with anti-6xHis and anti-mouse HRP conjugated antibodies (right panel). Aliquots of the preparation used for the activity measurements presented in the manuscript were used for this analysis. At this stage of the experiments, the protein solution was already frozen and thawed several times. Besides the major band of the expected ScpC6His monomer, some minor bands are seen. The Western blot identified the higher molecular weight bands as aggregates of ScpC6His and a minor band below the ScpC6His monomer as a degradation product. Traces of protein seen at about 16 kDa did not react with the antibody and represent either a N-terminal degradation product without His tag (the idea fits to the reduced size of the C-terminal fragment with His tag) or indeed an impurity.

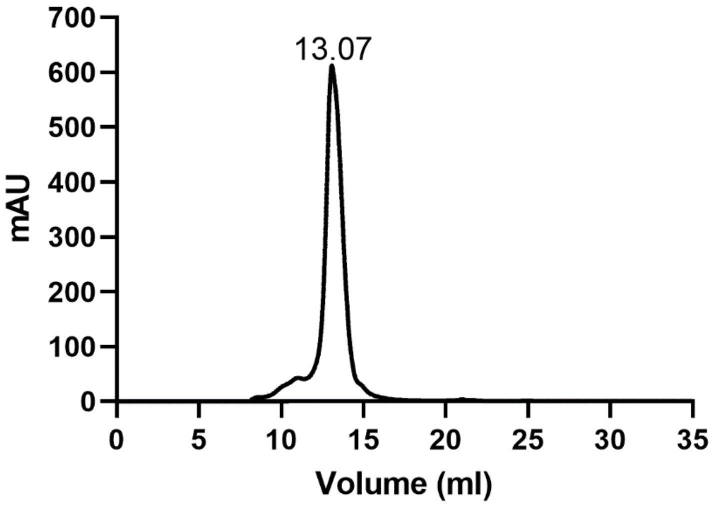

Figure S5. Gel filtration profile of ScpC purification. The Ni-NTA-purified ScpC was further analyzed via size exclusion chromatography using a Superdex 200 column (Cytiva) with an ÄKTA pure system (GE Healthcare Life Sciences). The elution profile revealed one symmetric peak at 13.07 ml (90.17% of the total peak aerea). The protein of the peak was confirmed to be ScpC by Western blot analysis. Based on column calibration, the protein in the peak fraction was estimated to have a molecular weight of about 135 kDa. Since the molecular weight of ScpC is 56.3 kDa, we propose that ScpC forms a homo dimer (molecular weight 112.6 kDa) under non-denaturing conditions. There is a small peak (9.3% of the total peak area) in front of the main peak. This peak originates most likely from higher oligomers or aggregates of ScpC similar as shown by SDS-PAGE and Western Blot analyses in Fig. S4. The proposed dimer formation is in agreement with crystal structures of other coenzyme A transferases that revealed the formation of homo dimers (e.g., PDB: 2OAS, 2G39, 5DDK).
